# Artificial intelligence in nursing: an integrative review of clinical and operational impacts

**DOI:** 10.3389/fdgth.2025.1552372

**Published:** 2025-03-07

**Authors:** Salwa Hassanein, Rabie Adel El Arab, Amany Abdrbo, Mohammad S. Abu-Mahfouz, Mastoura Khames Farag Gaballah, Mohamed Mahmoud Seweid, Mohammed Almari, Husam Alzghoul

**Affiliations:** ^1^ Nursing Department, Almoosa College of Health Sciences, Al Ahsa, Saudi Arabia; ^2^ Department of Community Health Nursing, Cairo University, Cairo, Egypt; ^3^ Health Informatics and Management Department, Almoosa College of Health Sciences, Al Ahsa, Saudi Arabia; ^4^ Faculty of Nursing, Beni-Suef University, Beni-Suef, Egypt

**Keywords:** artificial intelligence, nursing practice, clinical outcomes, operational efficiency, staff wellbeing, ethical implications

## Abstract

**Background:**

Advances in digital technologies and artificial intelligence (AI) are reshaping healthcare delivery, with AI increasingly integrated into nursing practice. These innovations promise enhanced diagnostic precision, improved operational workflows, and more personalized patient care. However, the direct impact of AI on clinical outcomes, workflow efficiency, and nursing staff well-being requires further elucidation.

**Methods:**

This integrative review synthesized findings from 18 studies published through November 2024 across diverse healthcare settings. Using the PRISMA 2020 and SPIDER frameworks alongside rigorous quality appraisal tools (MMAT and ROBINS-I), the review examined the multifaceted effects of AI integration in nursing. Our analysis focused on three principal domains: clinical advancements and patient monitoring, operational efficiency and workload management, and ethical implications.

**Results:**

The review demonstrates that AI integration in nursing has yielded substantial clinical and operational benefits. AI-powered monitoring systems, including wearable sensors and real-time alert platforms, have enabled nurses to detect subtle physiological changes—such as early fever onset or pain indicators—well before traditional methods, resulting in timely interventions that reduce complications, shorten hospital stays, and lower readmission rates. For example, several studies reported that early-warning algorithms facilitated faster clinical responses, thereby improving patient safety and outcomes. Operationally, AI-based automation of routine tasks (e.g., scheduling, administrative documentation, and predictive workload classification) has streamlined resource allocation. These efficiencies have led to a measurable reduction in nurse burnout and improved job satisfaction, as nurses can devote more time to direct patient care. However, despite these benefits, ethical challenges remain prominent. Key concerns include data privacy risks, algorithmic bias, and the potential erosion of clinical judgment due to overreliance on technology. These issues underscore the need for robust ethical frameworks and targeted AI literacy training within nursing curricula.

**Conclusion:**

This review demonstrates that AI integration holds transformative potential for nursing practice by enhancing both clinical outcomes and operational efficiency. However, to realize these benefits fully, it is imperative to develop robust ethical frameworks, incorporate comprehensive AI literacy training into nursing education, and foster interdisciplinary collaboration. Future longitudinal studies across varied clinical contexts are essential to validate these findings and support the sustainable, equitable implementation of AI technologies in nursing. Policymakers and healthcare leaders must prioritize investments in AI solutions that complement the expertise of nursing professionals while addressing ethical risks.

## 1 Introduction

Healthcare systems worldwide are undergoing transformative shifts, propelled by rapid advances in digital technologies and the burgeoning field of artificial intelligence (AI). AI, encompassing methodologies such as machine learning, natural language processing, and computer vision, holds the potential to enhance diagnostic accuracy, streamline clinical workflows, and support timely, evidence-based decision-making in multiple areas of care (1–3). Initially focused on specialty domains (e.g., radiology and oncology), AI's scope has broadened significantly, extending into nearly all spheres of patient management, including risk prediction, resource allocation, and patient engagement (4–6).

Within this evolving landscape, nursing—the largest healthcare workforce—assumes a pivotal role. Nurses serve as frontline providers, coordinating care, advocating for patients, and integrating complex streams of clinical data into actionable insights (7, 8). However, there remains a significant gap in understanding the comprehensive impact of AI on nursing practice, particularly in diverse healthcare settings and across various AI applications. As global demographic trends drive increases in patient acuity, comorbidities, and the prevalence of chronic diseases, nursing responsibilities continue to expand. These demands coincide with pressing workforce challenges, including nursing shortages, high turnover, and burnout, each of which threatens the quality and sustainability of care delivery (9–11). Research has consistently linked nurse staffing and well-being to patient outcomes, underscoring the need for interventions that can alleviate burdens, improve job satisfaction, and optimize patient care (12, 13).

### 1.1 Original contributions and novelty

This review is novel in synthesizing the integration of AI technologies into nursing practice, focusing on their impact on clinical outcomes, operational efficiency, and staff well-being. Unlike existing reviews that broadly explore AI in healthcare, this study specifically addresses nursing, emphasizing its critical role and unique challenges within healthcare systems.

AI-driven tools offer promising avenues to address these challenges. Predictive analytics may enable earlier detection of patient deterioration, machine learning algorithms could streamline triage and discharge planning, and natural language processing can reduce documentation burdens—all potentially freeing nurses to focus on activities that require clinical judgment and human empathy (14–16). For instance, wearable sensors integrated with AI can facilitate continuous patient monitoring, signaling subtle physiological changes before they manifest as complications (17). Such enhancements may support proactive, rather than reactive, nursing care. Beyond direct patient care, AI can also help manage administrative tasks and staffing logistics, ensuring that resources are deployed efficiently to maintain safe workloads and reduce burnout (18, 19).

A global perspective further highlights the need for careful AI integration. While many AI technologies are developed and tested in high-income settings, their adoption could help address healthcare gaps in resource-limited regions. For example, AI-driven decision support could guide nurses in remote or underserved areas, improving the consistency and accuracy of care where specialist expertise is scarce (20). Nonetheless, the existing literature predominantly focuses on high-income settings, indicating a need for more research in diverse and resource-constrained environments. Yet, translating such tools into diverse contexts demands sensitivity to local needs, infrastructure constraints, cultural factors, and workforce readiness. Without culturally congruent adaptation, AI risks becoming a tool that widens rather than bridges healthcare inequalities (21, 22).

As healthcare systems worldwide grapple with rising complexities and resource constraints, AI presents an opportunity to enhance patient outcomes, streamline operations, and support the nursing workforce. However, much remains unknown about the sustainability, scope, and ethical dimensions of AI's impact in real-world nursing contexts. Specifically, there is limited evidence on the long-term effects of AI integration, the variability of outcomes across different healthcare settings, and the ethical challenges posed by AI deployment in nursing. By systematically examining the existing evidence on AI integration in nursing practice, this review aims to provide a nuanced, evidence-based foundation for future innovation, ensuring that these technologies not only improve metrics of care, but also reinforce the humanistic and relational core of nursing.

### 1.2 Aim

To systematically evaluate the integration of AI technologies within nursing practice and assess their impact on clinical outcomes, operational efficiency, and the well-being of nursing staff.

### 1.3 Objectives

1. Evaluate the effectiveness of AI-driven tools in enhancing clinical diagnostics, therapeutic interventions, and patient monitoring, and determine their influence on the quality and outcomes of patient care.
2. Examine how AI integration optimizes operational workflows, manages nursing workloads, and mitigates staff burnout, thereby improving overall healthcare delivery and enhancing nursing job satisfaction.
3. Assess the ethical implications of AI integration in nursing practice.

## 2 Methodology

### 2.1 Study design

This integrative review adheres to the Preferred Reporting Items for Systematic Reviews and Meta-Analyses (PRISMA) 2020 guidelines to ensure comprehensive and transparent reporting (23). We selected the PRISMA framework for its well-established standards in systematic reviews, ensuring methodological rigor and transparency. SPIDER framework (Search, Phenomenon of Interest, Design, Evaluation, Research Type) was chosen for its effectiveness in capturing qualitative and mixed-methods research, which are pertinent to understanding the multifaceted impact of AI in nursing practice (24). The Mixed Methods Appraisal Tool (MMAT) was employed to evaluate the quality of diverse study designs (25), while the ROBINS-I (Risk Of Bias in Non-randomized Studies of Interventions) tool was utilized to assess the risk of bias specifically in non-randomized studies (26). This combination of methods was selected to accommodate the heterogeneous nature of the included studies and to provide a comprehensive appraisal of both quality and bias, thereby enhancing the overall robustness of our review.

### 2.2 SPIDER framework

The SPIDER tool was selected for its effectiveness in capturing qualitative and mixed-methods research, which are pertinent to understanding the multifaceted impact of AI in nursing practice. The components of SPIDER framework for this review are detailed in Table 1.

**Table 1:** SPIDER framework for study selection.

Component	Description
Sample	Registered nurses, nurse practitioners, and other nursing professionals across various settings.
Phenomenon of Interest	Integration of artificial intelligence (AI) technologies in nursing practice.
Design	Quantitative, qualitative, and mixed-methods studies.
Evaluation	Impact on clinical outcomes, operational efficiency, and nursing staff well-being.
Research Type	Empirical studies, including randomized controlled trials, cohort studies, cross-sectional studies, and qualitative studies.

### 2.3 Inclusion and exclusion criteria

To ensure the relevance and quality of the included studies, specific inclusion and exclusion criteria were established based on SPIDER framework (See Table 2: Inclusion and Exclusion Criteria).

**Table 2:** Inclusion and exclusion criteria.

Inclusion criteria	Exclusion criteria
Studies involving nurses or nursing staff utilizing AI technologies in clinical settings	Studies not involving nursing professionals or AI technologies
Integration of AI technologies in nursing practice, including diagnostic tools, therapeutic interventions, patient monitoring, workflow optimization, and staff well-being	Studies focusing solely on AI in other healthcare professions without nursing context
Quantitative studies (RCTs, cohort, cross-sectional), qualitative studies, and mixed-methods studies	Reviews, meta-analyses, editorials, commentaries, and non-empirical studies.
Clinical outcomes, operational efficiency, nursing workload, staff burnout, and job satisfaction	Studies not reporting on the specified outcomes
Publications in English	Non-English publications

### 2.4 Information sources and search strategy

A comprehensive literature search was conducted across multiple electronic databases to capture relevant studies published up to November 2024. The databases searched included PubMed, MEDLINE, CINAHL, EMBASE, Scopus, and IEEE Xplore. To minimize publication bias, an additional search for grey literature was also conducted using sources such as conference proceedings, dissertations, and preprint servers.

The search strategy was developed using a combination of keywords and Medical Subject Headings (MeSH) terms related to AI and nursing. Boolean operators (AND, OR) were employed to refine search results. The detailed search terms are presented in Table 3.

**Table 3:** Search terms used in the integrative review.

Concept	Keywords and MeSH terms
Artificial Intelligence	“Artificial Intelligence” OR “AI” OR “machine learning” OR “deep learning” OR “natural language processing” OR “computer vision”
Nursing	“Nursing” OR “Registered Nurse” OR “Nurse Practitioner” OR “Clinical Nurse” OR “Nursing Staff”
Integration	“Integration” OR “Implementation” OR “Adoption” OR “Utilization”
Outcomes	“Clinical Outcomes” OR “Operational Efficiency” OR “Workload Management” OR “Burnout” OR “Staff Well-being”

### 2.5 Selection process

**Figure 1:**
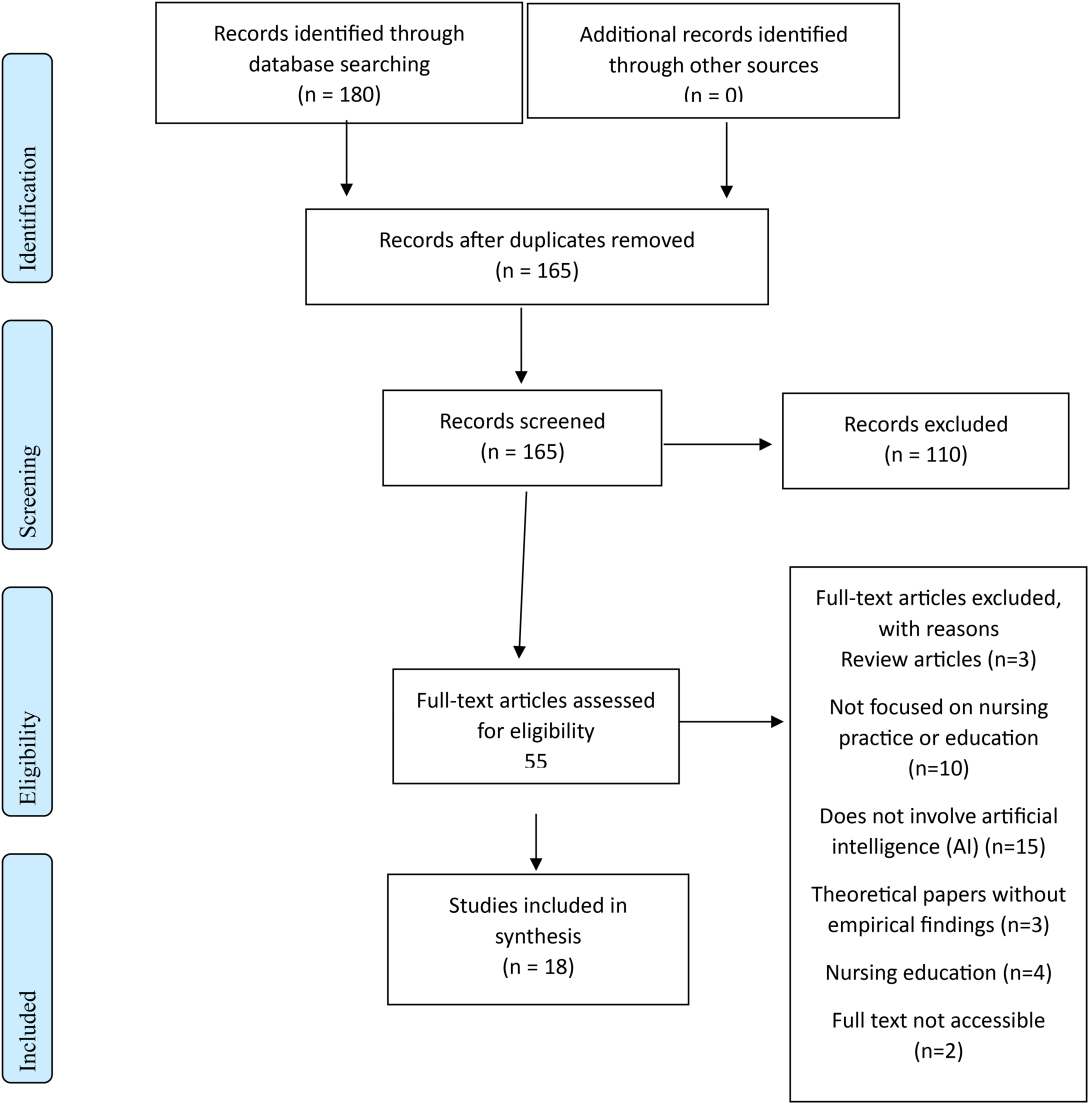
PRISMA flow diagram.

The records identified through database searches were imported into Rayyan, a systematic review screening tool (27). Two independent reviewers conducted the screening process to minimize bias, with discrepancies resolved through discussion or consultation with a third reviewer. Titles and abstracts of identified studies were screened against the inclusion and exclusion criteria. Full-text articles of potentially relevant studies were retrieved and assessed for eligibility based on the predefined criteria. The PRISMA flow diagram (Figure 1) below illustrates the study selection process.

### 2.6 Data extraction

Data extraction was performed independently by two reviewers using a standardized data extraction form. The extracted data included study characteristics [author(s), year of publication, country, study design], population details (sample size, demographic information, nursing roles, AI technologies), outcomes (clinical outcomes, operational efficiency metrics, staff well-being measures), and key findings. Additionally, more detailed definitions of specified outcomes were incorporated: Clinical Outcomes: Refers to measurable changes in patient health status resulting from nursing interventions, including diagnostic accuracy, therapeutic effectiveness, and patient quality of life. Operational Efficiency: Encompasses the optimization of nursing workflows, resource allocation, and reduction of administrative burdens to enhance overall healthcare delivery. Staff Well-being: Involves the physical, mental, and emotional health of nursing staff, including measures of job satisfaction, burnout levels, and mental health support.

Additionally, the data extraction process was refined to enhance clarity. In cases of disagreements in data extraction beyond involving a third reviewer, such as persistent discrepancies or complex interpretations, a consensus meeting was held where both reviewers discussed the discrepancies in detail to reach an agreement. If consensus could not be achieved, a fourth reviewer was consulted to make the final decision. This additional step ensured that data extraction was accurate and reliable. Discrepancies in data extraction were resolved through discussion or by involving a third reviewer.

### 2.7 Quality assessment and risk of bias

The ROBINS-I tool was utilized to assess the risk of bias (26). ROBINS-I is specifically designed for non-randomized studies of interventions and evaluates bias across seven domains: confounding, selection of participants, classification of interventions, deviations from intended interventions, missing data, measurement of outcomes, and selection of reported results.

To comprehensively appraise the methodological quality of the included studies, MMAT was employed (28). MMAT is adept at evaluating a diverse range of study designs, including quantitative, qualitative, and mixed-methods research. The tool assesses studies across five critical domains: clarity and relevance of research questions, appropriateness of sampling strategies, representativeness of the sample, validity and reliability of measurements, and adequacy of response or follow-up rates.

## 3 Results

### 3.1 Study characteristics

This integrative review included 18 studies conducted between 2020 and 2024 across various countries, including Saudi Arabia, China, Bangladesh, South Korea, Norway, Canada, the United Kingdom, Egypt, Germany, and Brazil. The studies employed diverse research designs, such as descriptive cross-sectional studies, randomized controlled trials (RCTs), qualitative analyses, exploratory quantitative and qualitative research, quasi-experimental studies, prospective controlled studies, interpretive phenomenological analyses, cross-sectional online surveys, and retrospective observational studies. The populations studied encompassed registered nurses from governmental hospitals, COPD patients in emergency care, nursing professionals in tertiary hospitals, orthopedic and surgical patients, postpartum women, critical care nurse leaders in ICUs.

AI interventions varied widely, including online surveys to assess AI awareness, AI-assisted postoperative follow-up systems, AI-enhanced diagnostic imaging methods, mobile applications for reducing nurse burnout, machine learning algorithms for predictive workload classification, AI-based nutritional nursing models, pelvic floor rehabilitation training using AI-processed imaging, and AI systems for pain monitoring in NICUs. The review captures the comprehensive impact of AI on both patient health outcomes and the operational aspects of nursing practice. This holistic approach ensures that the multifaceted effects of AI integration are thoroughly examined, encompassing improvements in diagnostic accuracy, therapeutic interventions, and patient monitoring, as well as enhancements in operational efficiency and nursing staff well-being (See Supplementary Table S1: Included studies summary).

### 3.2 Risk of bias

We evaluated the risk of bias of all 18 included studies using the ROBINS-I tool (26). Overall, the 18 studies were rated as having a moderate risk of bias (29–48). The primary drivers of moderate bias were incomplete adjustment for confounders (particularly baseline health differences, demographic factors, and prior AI exposure), reliance on subjective or self-reported outcome measures, and selective reporting due to absent or unreferenced protocols. While selection bias was generally low because most studies had well-defined inclusion criteria, a few relied on convenience sampling, which may limit the generalizability of their findings. Interventions were generally well classified, adherence to intervention protocols was high, and missing data were commonly addressed with appropriate statistical methods. In light of these findings, most studies should be interpreted cautiously, highlighting the need for more robust methodological designs and comprehensive confounder control in future research (See Supplementary Table S2).

### 3.3 Quality assessment MMAT

The quality of the 18 included studies was systematically evaluated using the MMAT (49). Most of the included studies demonstrated high methodological quality, with 15 studies meeting all five MMAT criteria. These studies showcased clear and well-defined research questions aligned with their objectives, employed appropriate sampling strategies, and used validated and reliable measurement tools. They also achieved high response or follow-up rates, ensuring data completeness and reliability. For instance, RCTs consistently implemented rigorous designs with randomization procedures, baseline comparability, and objective outcome measures, as seen in studies evaluating AI-assisted interventions in clinical settings or nursing education.

However, three studies did not meet all five MMAT criteria. One descriptive study assessing nurses’ awareness and attitudes toward AI in clinical practice met four criteria due to a limited sample drawn from three hospitals, despite the use of validated tools and an adequate response rate (40). Similarly, an RCT evaluating the application of AI in emergency nursing for COPD patients failed to provide sufficient details on randomization procedures and generalizability, meeting only three criteria (32). Lastly, a cross-sectional survey exploring nurses’ perceptions and knowledge of AI met four criteria, as its convenience sampling strategy and relatively small sample size limited generalizability beyond the study's regional context (30). These limitations suggest that while the findings from these studies are informative, they should be interpreted with caution. The moderate risk of bias in these studies highlights the need for more robust research designs in future studies to confirm and expand upon these initial findings. Integrating the insights from these studies with those of higher-quality studies provides a more comprehensive understanding of the current landscape of AI integration in nursing practice, albeit with an acknowledgment of the methodological constraints present in some of the included research.

The validity and reliability of measurements were a consistent strength across the included studies. Quantitative research utilized standardized tools, such as the MSK-HQ, PEMAT, and Copenhagen Burnout Inventory, ensuring robust and comparable results. Qualitative studies adopted rigorous data collection methods, including semi-structured interviews and thematic analysis, with some integrating AI-based analytical tools to enhance insight generation. These approaches contributed to the reliability and depth of the findings.

Sampling strategies were generally appropriate for the study designs, with many RCTs employing robust randomization techniques to minimize bias. Nevertheless, limitations were noted in some descriptive and qualitative studies that relied on convenience sampling, which may introduce selection bias. Generalizability was occasionally restricted due to small sample sizes or single-center designs, as observed in a few studies.

In conclusion, the majority of the included studies demonstrated high methodological rigor, meeting all five MMAT criteria. These studies provided robust evidence of the application of AI in nursing and healthcare, with clear research questions, appropriate sampling strategies, validated measurement tools, and reliable outcomes. While three studies fell short in specific domains, their findings remain valuable (See Supplementary Table S3).

### 3.4 Thematic synthesis

This integrative review synthesizes findings from 18 studies evaluating the integration of AI technologies into nursing practice, providing a comprehensive analysis of their impact on clinical outcomes, operational efficiency, nursing staff well-being, and ethical and safety implications. Thematic analysis revealed three primary themes: Clinical and Therapeutic Advancements through AI Integration, Operational Optimization and Staff Support, and Ethical Implications. To ensure a thorough exploration, we have delved deeper into each theme, examining the specific AI applications, their effectiveness, and the challenges encountered during implementation (See Table 4 for the Key Findings).

**Table 4:** Alignment of study objectives with findings and themes.

Objective	Key findings	Themes
1. Evaluate the effectiveness of AI-driven tools in enhancing clinical diagnostics, therapeutic interventions, and patient monitoring, and determine their influence on the quality and outcomes of patient care.	AI tools improved diagnostic accuracy, therapeutic interventions, and real-time patient monitoring, leading to better patient outcomes and personalized care.	Clinical and therapeutic advancements through AI integration
2. Examine how AI integration optimizes operational workflows, manages nursing workloads, and mitigates staff burnout, thereby improving overall healthcare delivery and enhancing nursing job satisfaction.	AI technologies streamlined workflows, automated administrative tasks, optimized resource use, and reduced nurse workload and burnout while improving job satisfaction.	Operational optimization and staff support
3. Assess the ethical implications of AI integration in nursing practice.	Identified concerns related to data privacy, algorithmic bias, and overreliance on AI, highlighting the need for ethical frameworks and training.	Ethical implications

### 3.5 Clinical and therapeutic advancements through AI integration

AI technologies have significantly enhanced the precision of clinical diagnostics, the effectiveness of therapeutic interventions, and the capacity for continuous patient monitoring. These advancements facilitate earlier and more accurate identification of medical conditions, enable personalized care strategies, and improve overall patient outcomes.

#### 3.5.1 Enhanced diagnostic precision and therapeutic interventions

AI integration in nursing practice significantly improved diagnostic accuracy and therapeutic interventions. For instance, Jiang et al. (35) demonstrated that AI-based FCM algorithms enhanced MRI diagnostics for ovarian endometriosis, increasing accuracy from 63.15% (conventional MRI) to 94.32%. This improvement not only aids nurses in making more informed clinical decisions but also leads to better patient outcomes, such as higher satisfaction rates and reduced complications. Similarly, Xu et al. (46) integrated the Otsu method algorithm into indocyanine green angiography (ICGA) for diagnosing intracranial aneurysms, which significantly improved diagnostic precision, reduced complications, and enhanced patient satisfaction.

In the realm of therapeutic interventions, Zhang et al. (29) utilized AI-enhanced MRI in pelvic floor muscle rehabilitation for rectal cancer patients, resulting in improved image clarity and diagnostic precision. This advancement allowed nurses to tailor rehabilitation programs more effectively, enhancing postoperative recovery and patient quality of life. Du et al. (41) implemented an AI-based Plan-Do-Check-Action (PDCA) home nursing model for patients with diabetic nephropathy, which led to significant improvements in clinical effectiveness and patient satisfaction over 12 months by enabling dynamic adjustments to care plans based on AI-generated insights.

#### 3.5.2 Real-time patient monitoring and early intervention

AI-integrated monitoring systems provide continuous, real-time data, enabling nurses to detect clinical changes early and intervene promptly, thereby improving patient safety and care quality. In emergency nursing for COPD patients, Hong et al. (32) found that AI-driven telemedicine platforms facilitated real-time patient monitoring and early intervention, which reduced hospitalization rates and enhanced patients’ quality of life. These AI tools empower nurses to make timely decisions, thereby improving both operational efficiency and patient care quality. Liu et al. (34) evaluated the iThermonitor WT705, an AI-assisted wearable thermometer, in surgical wards. The device identified fevers in 22% of patients compared to 17% with standard mercury thermometers and detected fevers up to 4.35 h earlier, allowing nurses to manage potential infections or complications proactively.

Racine et al. (42) assessed AI for pain monitoring in neonatal intensive care units (NICUs). The AI-assisted systems enhanced the accuracy and consistency of pain assessments, enabling more effective pain management strategies for preterm infants. While these findings are promising, it is essential to consider the scalability and long-term sustainability of such AI systems in various clinical settings. Nurses utilized these tools to standardize pain assessments, ensuring timely and appropriate interventions, which improved patient comfort and reduced the risk of long-term developmental issues associated with unmanaged pain.

### 3.6 Operational optimization and staff support

AI technologies have optimized nursing workflows, resource allocation, and administrative tasks, thereby enhancing operational efficiency and reducing the burden on nursing staff. These optimizations are crucial for maintaining high standards of patient care while ensuring that nursing professionals are supported, and their well-being is preserved.

#### 3.6.1 Workflow efficiency and resource allocation

AI-driven predictive models and automation tools have streamlined nursing workflows and optimized the allocation of resources, ensuring efficient use of nursing personnel and enhancing patient care delivery. Rosa et al. (44) developed an AI-based nursing workload classifier that achieved 72% accuracy and 82% AUC-ROC. By automating workload assessments, this tool allows nurses to manage their time more effectively, reducing administrative burdens and enabling a focus on patient-centered care. Consequently, patients receive more timely and personalized interventions, enhancing overall healthcare delivery. Seibert et al. (48) highlight the potential of AI to optimize staff scheduling and skill mix, improve route planning for home care services, and aid in institutional workflow management.

Similarly, Chen et al. (47) implemented an AI-based “Internet + Hospital-to-Home” (H2H) nutritional nursing model for chronic kidney disease (CKD) patients. This model optimized nutritional assessments and monitoring through automated data analysis and personalized care plans, reducing manual assessment time and enhancing resource allocation based on real-time patient needs. These applications demonstrate how AI can facilitate informed decision-making in resource distribution, thereby enhancing operational efficiency and ensuring that nursing resources are utilized effectively.

#### 3.6.2 Staff well-being and burnout mitigation

AI-based interventions have been effective in reducing nurse burnout and enhancing staff well-being, thereby supporting nurse retention and overall job satisfaction. Cho et al. (36) evaluated the Nurse Healing Space app, an AI-based mobile intervention designed to reduce burnout. The app significantly decreased burnout scores, job stress, and stress responses among nurses while increasing user satisfaction through personalized recommendations. The intervention included tailored programs such as mindfulness meditation, laughter therapy, storytelling and reflective writing, and acceptance and commitment therapy, each targeting different dimensions of burnout. Nurses engage with the app to participate in stress-reduction activities, thereby enhancing their well-being and job satisfaction.

Moreover, the integration of AI tools for workload management and administrative automation indirectly contributes to staff well-being by reducing the administrative burden on nurses. Bian et al. (37) evaluated an AI-assisted postoperative follow-up system for orthopedic patients, which reduced time spent on follow-ups through automation. This efficiency not only streamlines nursing workflows but also allows nurses to dedicate more time to direct patient care, thereby improving patient engagement and satisfaction.

### 3.7 Ethical implications

The integration of AI into nursing practice introduces several ethical and safety considerations that must be carefully addressed to ensure responsible and equitable implementation. This theme encompasses concerns related to data privacy, algorithmic bias, overreliance on AI systems, job roles, patient autonomy, and overall patient safety. Additionally, the ethical framework guiding AI implementation must consider the diverse socio-cultural contexts in which nursing operates.

#### 3.7.1 Data privacy and security

Data privacy and security are paramount concerns in AI integration. Studies by Hong et al. and Rony et al. (32, 38) highlighted significant apprehensions regarding the extensive collection and use of personal health data by AI systems. Nurses called for clear guidelines and frameworks to govern AI's ethical implementation in healthcare to protect patient information and maintain trust in healthcare systems. The implementation of robust data encryption and access control measures is essential to safeguard sensitive patient information from potential breaches and misuse. Seibert et al. (48) highlight barriers such as limited data digitization, privacy and security concerns, and the complexity of nursing tasks that restrict AI to simpler functions.

The risk of algorithmic bias is a critical ethical concern that can lead to unequal treatment outcomes. Racine et al. (42) emphasized the need for auditing algorithms and resolving potential disagreements between AI and human judgments to ensure fairness and equity in patient care. Addressing algorithmic bias is essential to prevent disparities in healthcare delivery and to ensure that AI tools provide unbiased support to all patient populations. Ongoing monitoring and iterative testing of AI algorithms are necessary to identify and mitigate biases that may arise from skewed training data or inherent design flaws.

#### 3.7.2 Overreliance on AI and clinical judgment

While AI enhances decision-making, there is a valid concern about overreliance on AI systems, potentially eroding critical thinking and clinical expertise among nurses. Hassan and El-Ashry (31) reported that nurses may become overly dependent on AI recommendations, risking the diminution of their clinical judgment and decision-making skills. This overreliance can lead to a decrease in the ability to identify nuanced patient needs that require human intuition and empathy, thereby impacting the quality of patient care. Sommer et al. (30) found that despite limited AI knowledge among nurses, the majority view AI positively, recognizing its potential to alleviate workload pressures and improve nursing efficiency. However, concerns about depersonalization, errors, and job displacement remain barriers to broader acceptance. This highlights the critical need for nursing education and training to fully leverage AI-driven diagnostic tools effectively.

## 4 Discussion

The findings of this integrative review underscore the transformative potential of AI in nursing practice. By synthesizing data from 18 studies, three critical themes emerge: Clinical and Therapeutic Advancements through AI Integration, Operational Optimization and Staff Support, and Ethical and Safety Implications. While these themes highlight significant advancements and benefits, our critical analysis reveals persistent challenges such as data privacy concerns, algorithmic biases, and the potential erosion of clinical judgment. Furthermore, the variability in AI implementation across different healthcare settings indicates a need for standardized protocols and comprehensive training programs. Our review identifies these gaps and emphasizes the necessity for future research to address these issues, thereby contributing original insights and recommendations to the field.

Limited studies in low- and middle-income countries (LMICs) restrict the generalizability of findings globally. Most of the existing research focuses on high-income settings, leaving a significant gap in understanding how AI integration affects nursing practices in resource-constrained environments. Scarcity of research on the long-term sustainability of AI tools in nursing practice poses challenges in assessing the enduring impact and viability of these technologies. Additionally, there is insufficient exploration of the impact of AI on nurse-patient relationships, particularly concerning empathy and compassionate care. While AI can enhance operational efficiency and clinical outcomes, its influence on the humanistic aspects of nursing care remains underexplored, raising concerns about the potential depersonalization of patient interactions.

### 4.1 Clinical and therapeutic advancements through AI integration

The integration of AI technologies has significantly advanced the precision and reliability of nursing assessments and interventions. AI-driven diagnostic tools have demonstrated remarkable improvements in diagnostic accuracy and therapeutic effectiveness across various clinical settings. This finding aligns with Armoundas et al. (50), who reported that AI-enhanced cardiac imaging tools, such as echocardiography and cardiac MRI, automated key diagnostic tasks and democratized cardiac expertise, thereby reducing disparities in resource-limited settings. These advancements enable nurses to integrate high-quality imaging data into patient care plans, enhancing the timeliness and accuracy of diagnoses.

Similarly, Yasmin et al. (51) demonstrated that neural network algorithms achieved an 85% accuracy rate in diagnosing heart failure by integrating clinical and demographic data. The study emphasized AI's ability to detect heart failure up to six months before clinical diagnosis, allowing nurses to implement preventive care strategies. In our review, Xu et al. (46) showed how AI-assisted imaging optimized perioperative care, reducing surgical complications and improving postoperative outcomes. These findings are corroborated by Tafat et al. (52), who noted the efficacy of AI-powered wearable sensors in enhancing post-surgical rehabilitation by monitoring progress and personalizing therapy plans, thereby empowering nurses to deliver tailored interventions.

AI's role extends beyond diagnostics to therapeutic interventions. Zhang et al. (29) utilized AI-enhanced MRI in pelvic floor muscle rehabilitation for rectal cancer patients, resulting in improved image clarity and diagnostic precision. This advancement allowed nurses to tailor rehabilitation programs more effectively, enhancing postoperative recovery and patient quality of life. Du et al. (41) implemented an AI-based PDCA home nursing model for patients with diabetic nephropathy, which led to significant improvements in clinical effectiveness and patient satisfaction over 12 months by enabling dynamic adjustments to care plans based on AI-generated insights. However, it is crucial to recognize that these advancements are contingent upon the availability of high-quality data and the integration of AI systems within existing healthcare infrastructures. These advancements collectively highlight AI's capacity to enhance both diagnostic and therapeutic dimensions of nursing care, leading to improved patient outcomes and more personalized care strategies.

### 4.2 Operational optimization and staff support

AI technologies have demonstrated transformative potential in optimizing nursing workflows, resource allocation, and administrative efficiency. Predictive models, such as those developed by Rosa et al. (44), have significantly improved staff allocation by identifying workload determinants and ensuring adequate coverage in high-demand areas. This finding is echoed by Epelde (53), who reported that AI-powered predictive analytics not only optimize staff scheduling but also enhance resource utilization by forecasting bed occupancy and patient flow, thereby reducing operational bottlenecks and improving patient outcomes.

AI-driven process automation further alleviates the burden of manual administrative tasks. Bian et al. (37) found that AI-assisted follow-up systems for postoperative care achieved comparable effectiveness to manual methods while drastically reducing time and resource demands. These efficiencies are consistent with Varnosfaderani et al. (6), who emphasized the role of AI in streamlining hospital operations, from billing to electronic health record analysis. Automating routine tasks enable nurses to dedicate more time to direct patient care, thereby improving care quality and patient satisfaction.

Beyond administrative efficiencies, AI also contributes to clinical safety and operational reliability. AI-powered early warning systems (EWS) have proven effective in real-time monitoring and early detection of critical conditions, including sepsis and acute kidney injury (53). These systems reduce ICU admissions and mortality rates by facilitating timely interventions, reinforcing the importance of AI in enhancing patient safety and reducing nurse workload. Nevertheless, the successful implementation of EWS requires seamless integration with existing clinical workflows and continuous training for nursing staff to effectively interpret and act upon AI-generated alerts. The economic implications of AI adoption are equally compelling. By preventing adverse events, improving infection control, and optimizing resource allocation, AI generates cost savings while maintaining high-quality care (53). The integration of predictive analytics in managing nosocomial infections—such as through antimicrobial stewardship programs—further underscores AI's role in reducing hospital-acquired complications and enhancing operational efficiency.

However, challenges remain in achieving seamless AI integration into clinical workflows. Data quality issues, ethical considerations, and clinician apprehension toward over-reliance on AI tools are notable barriers. Addressing these challenges requires robust training programs, as suggested by Sommer et al. (30), and efforts to standardize data practices to improve interoperability and reliability (6). Additionally, the financial costs associated with AI implementation and maintenance must be carefully managed to ensure sustainability, especially in resource-limited settings. Effective integration also necessitates interdisciplinary collaboration to ensure that AI tools are user-friendly and aligned with nursing workflows, thereby fostering trust and enhancing the adoption of AI technologies in clinical settings.

### 4.3 Ethical and safety implications

The integration of AI into nursing practice introduces several ethical and safety considerations that must be carefully addressed to ensure responsible and equitable implementation. This theme encompasses concerns related to data privacy, algorithmic bias, overreliance on AI systems, job roles, patient autonomy, and overall patient safety. Furthermore, ensuring that AI tools are developed and implemented with ethical standards that respect patient rights and promote equity is essential for sustainable adoption.

### 4.4 Staff well-being and burnout management

AI-based interventions have shown considerable promise in mitigating nurse burnout and enhancing job satisfaction. Cho et al. (36) demonstrated that the Nurse Healing Space app led to significant reductions in burnout scores among nurses. This finding aligns with Dailah et al. (54), who highlighted the role of AI-powered chatbots, such as Wysa and Woebot, in providing 24/7 mental health support through cognitive behavioral therapy (CBT) and mindfulness practices. These tools not only address stress and anxiety but also offer predictive analytics to identify nurses at risk of mental health issues, enabling early intervention and tailored support.

AI-driven operational enhancements also contribute to improved well-being among nursing staff. Varnosfaderani et al. (6) emphasized the role of AI in optimizing workflows through automated scheduling and staffing systems, which reduce conflicts and streamline decision-making processes. This optimization alleviates the administrative burden on nurses, allowing them to focus more on direct patient care, thereby fostering job satisfaction and professional fulfillment.

The transformative potential of AI in addressing burnout extends beyond individual interventions. By leveraging tools such as predictive analytics and wearable devices, AI empowers nurses to manage their workloads more effectively, promoting resilience and well-being. However, achieving this potential requires addressing barriers such as algorithmic biases, data privacy concerns, and resistance to AI adoption due to knowledge gaps (54). Training programs and interdisciplinary collaboration are essential to foster trust and enhance the effective use of AI in nursing practice.

Balancing technological advancements with the need for compassionate, patient-centered care will be critical to ensuring the ethical and sustainable integration of AI in nursing practice. AI holds significant promise in improving nurse well-being and mitigating burnout through targeted mental health interventions, operational efficiency, and workload management. Nonetheless, ensuring that these tools are implemented thoughtfully and ethically will maximize their benefits while minimizing potential drawbacks. This balance is crucial to maintaining the humanistic and relational core of nursing, which is fundamental to high-quality patient care.

### 4.5 Implications

The integration of AI into nursing practice presents profound and far-reaching implications that extend across clinical practice, healthcare policy, education, and research. Clinically, AI technologies have the potential to revolutionize patient care by enhancing diagnostic accuracy, personalizing therapeutic interventions, and streamlining clinical workflows, thereby elevating the overall quality of care and patient outcomes. Operationally, AI-driven automation of administrative tasks and optimized resource allocation can significantly alleviate the burdens on nursing staff, reducing burnout and enhancing job satisfaction, which is critical for maintaining a sustainable and resilient nursing workforce. From a policy perspective, the ethical integration of AI necessitates the development of comprehensive governance structures that address data privacy, algorithmic transparency, and equitable access, ensuring that AI implementations comply with ethical standards and protect patient rights. Educationally, the incorporation of AI literacy into nursing curricula is essential for preparing the next generation of nurses to effectively engage with and leverage AI technologies, ensuring that they are competent in utilizing these tools to enhance patient care. Additionally, continuous education and training programs are vital to keep nursing professionals updated with the latest advancements in AI, fostering a culture of lifelong learning and adaptability within the profession. Research-wise, the findings underscore the necessity for ongoing, high-quality research that explores the long-term effects of AI integration on clinical outcomes, operational efficiency, and nursing staff well-being. Expanding research efforts to include diverse cultural and geographical contexts, particularly in resource-limited settings, will enhance the generalizability of findings and support the development of adaptable AI solutions tailored to various clinical environments. These implications highlight the transformative potential of AI in nursing, emphasizing the need for strategic, ethical, and informed approaches to its implementation to achieve scalable and sustainable improvements in healthcare delivery.

### 4.6 Recommendations

To effectively harness the transformative potential of AI in nursing practice and address the multifaceted challenges identified in this review, it is imperative to establish comprehensive and ethically grounded frameworks that govern AI deployment within healthcare settings. Policymakers and healthcare institutions must collaborate to develop robust guidelines addressing data privacy, algorithmic transparency, and bias mitigation, ensuring that AI technologies are utilized equitably and responsibly. Integrating AI literacy into nursing education curricula is essential; nursing programs should incorporate modules that equip future nurses with the competencies necessary to effectively utilize AI-driven tools, fostering a generation of digitally proficient healthcare professionals. Additionally, continuous professional development opportunities should be provided to existing nursing staff to facilitate ongoing adaptation to evolving AI technologies. Strategic integration of AI into clinical workflows is another critical recommendation. Engaging nurses and other frontline healthcare workers in the design and implementation phases of AI tools will ensure these technologies are intuitive, user-friendly, and seamlessly aligned with clinical practices, thereby enhancing adoption and effectiveness. Investment in high-quality, diverse data infrastructure is necessary to support the training and validation of AI systems, ensuring that datasets are representative and minimize biases, thus improving the generalizability of AI applications across diverse patient populations. Furthermore, fostering interdisciplinary collaboration is paramount; partnerships between nurses, data scientists, ethicists, and IT professionals will ensure that AI solutions are comprehensive, ethically sound, and tailored to meet the nuanced needs of clinical environments. Lastly, promoting robust research initiatives that focus on longitudinal studies and diverse healthcare settings will generate critical evidence on the long-term impacts of AI integration, informing best practices and guiding future innovations in nursing practice. By implementing these strategic recommendations, healthcare systems can effectively scale up AI integration, fostering innovative and impactful approaches that enhance patient care, optimize operations, and support the well-being of nursing professionals. These measures will not only address the current challenges but also pave the way for a resilient and technologically adept nursing workforce poised to meet the evolving demands of modern healthcare.

### 4.7 Strengths and limitations

Adherence to the PRISMA 2020 guidelines and the utilization of SPIDER framework ensured a structured and transparent study selection process, enhancing the reliability and reproducibility of the findings. Additionally, the application of the ROBINS-I tool for risk of bias assessment and the MMAT for quality evaluation provided a robust appraisal of the included studies, ensuring that the synthesized evidence is both valid and reliable. However, this review is not without limitations. The exclusion of non-English publications introduces potential language bias, potentially omitting relevant studies conducted in non-English-speaking regions, which may limit the comprehensiveness and generalizability of the findings. The heterogeneity of AI technologies and the variability in healthcare settings among the included studies introduce significant challenges in synthesizing findings and drawing uniform conclusions. This diversity, while reflective of the dynamic nature of AI integration, poses challenges for identifying consistent patterns and generalizable outcomes.

## 5 Conclusion

This integrative review highlights the transformative impact of AI in nursing practice, revealing significant advancements in clinical diagnostics, therapeutic interventions, and operational efficiencies. AI-driven tools enhance patient care quality, optimize resource allocation, and support nursing staff well-being, addressing critical workforce challenges such as burnout and job dissatisfaction. However, the integration of AI also presents ethical challenges, including data privacy concerns, algorithmic bias, and the potential erosion of clinical judgment. To maximize the benefits of AI while mitigating its risks, it is essential to develop comprehensive ethical frameworks, integrate AI literacy into nursing education, and foster interdisciplinary collaboration. Investing in high-quality, diverse data infrastructure and promoting robust, longitudinal research will further support the sustainable and equitable implementation of AI in nursing. By embracing these strategies, healthcare systems can leverage AI to advance patient-centered, efficient, and compassionate care, ultimately enriching the nursing profession and enhancing healthcare delivery globally.
