# Supplementary material for: Artificial intelligence in nursing: an integrative review of clinical and operational impacts
Source: Front Digit Health. 2025 Mar 7;7:1552372. doi: 10.3389/fdgth.2025.1552372 (PMC11926144; doi:10.3389/fdgth.2025.1552372)
Supplement: Supplementary file 1 [file Table1.pdf]

Supplementary Table 1: Included studies summary

| Study ID | Year | Country | Study Design | Population/Setting | AI Intervention | Impact on Clinical Outcomes | Impact on Operational Efficiency\ workload | Impact on Nursing Staff Well-being, burnout | Key Findings | Limitations |
|---|---|---|---|---|---|---|---|---|---|---|
| Alruwaili et al. | 2024 | Saudi Arabia | Descriptive Cross-Sectional | 220 registered nurses from three governmental hospitals in Jouf region | Online survey assessing awareness and attitudes toward AI tools in clinical practice | Moderate awareness; 58.2% used AI in healthcare | N/A | N/A | Positive attitudes among younger and educated nurses; concerns about privacy, job roles, and errors | Limited generalizability; cross-sectional design; convenience sampling |
| Hong et al. | 2021 | China | Randomized Controlled Trial (RCT) with Sub-Analyses | 447 COPD patients in emergency care; sub-cohorts of 101 and 29 | AI-based medical interventions: web-based knowledge exercises, telemedicine | Improved quality of life at 12 months; reduced hospitalization rates and length of stay in AI groups | Reduced time and resource consumption in follow-ups | N/A | Significant QoL improvements after 12 months; reduced hospitalization rates and stay lengths in AI groups | High dropout rate due to internet issues; small sample size in sub-cohorts |
| Rony et al. | 2024 | Bangladesh | Descriptive Qualitative Study | 23 nursing professionals at three tertiary hospitals in Dhaka | Exploration of perspectives on AI integration in nursing care | Enhanced patient outcomes through AI support | Potential for optimized workflows with AI | Highlighted need for training and adaptability | Optimism towards AI for enhancing care; concerns on training, ethics, and workflow integration | Small sample size; single region; no patient or AI developer perspectives included |
| Bian et al. | 2020 | China | Exploratory Quantitative and Qualitative Study | 270 orthopedic patients (AI-assisted follow-up) vs. 2656 patients (manual follow-up) | AI-assisted postoperative follow-up system vs. manual follow-up | Similar clinical effectiveness to manual follow-up; higher feedback rates in AI group | Significantly reduced time and resource consumption | N/A | AI-assisted follow-up achieved similar effectiveness with higher feedback rates and lower time costs; feedback depth limited | Short probation period for AI system; no integration with additional follow-up methods like chatbots |
| Xu et al. | 2022 | China | Quasi-Experimental Study | 86 intracranial aneurysm patients undergoing craniotomy clipping | AI-enhanced cerebral angiography (Otsu method) combined with perioperative nursing intervention | Improved surgical outcomes; reduced complications; enhanced quality of life | N/A | Higher nursing satisfaction rates | AI-enhanced ICGA significantly improved treatment outcomes, reduced complications, and enhanced QoL compared to standard care | Small sample size; short postoperative follow-up; limited to a single center |

| | | | | | | | | | | |
|---|---|---|---|---|---|---|---|---|---|---|
| Cho et al. | 2024 | South Korea | Single-Arm Trial | 300 nurses experiencing burnout | AI-based tailored mobile intervention using "Nurse Healing Space" app | Significant reduction in burnout levels | N/A | Improved mental health and job satisfaction | Significant reduction in burnout, job stress, and stress responses; satisfaction increased with optimized AI recommendations | Lack of control group; limited generalizability due to specific sample; ongoing algorithm optimization |
| Jiang et al. | 2022 | China | Comparative Experimental Study | 116 patients with ovarian endometriosis | MRI diagnosis using AI-based FCM algorithm and comprehensive nursing intervention | Enhanced diagnostic accuracy; improved nursing satisfaction | N/A | Reduced adverse reactions and improved satisfaction | AI-FCM algorithm significantly improved diagnostic accuracy, Dice coefficient, and specificity; comprehensive nursing reduced adverse reactions and improved satisfaction | Limited to a single hospital; no long-term follow-up on patient outcomes |
| Marcuzzi et al. | 2023 | Norway | Randomized Clinical Trial | 294 patients with neck and/or low back pain referred to specialist care | AI-based SELFBACK app for self-management support vs. usual care and web-based e-Help intervention | No significant improvement in musculoskeletal health | Drastically reduced time and resource demands | N/A | AI-based app did not significantly improve musculoskeletal health over usual care or web-based intervention; higher global perceived effect | Low engagement with the app; no significant long-term improvement; generalizability limited to similar care settings |
| Liu et al. | 2020 | China | Prospective Comparative Study | 526 surgical patients in surgical wards at a university hospital | Continuous temperature monitoring using AI-based wearable device (iThermonitor WT705) vs. mercury thermometer | Earlier fever detection; higher peak temperature recording | Continuous monitoring feasible; reduced manual checks | N/A | iThermonitor showed acceptable accuracy, earlier fever detection, and higher peak temperature recording; high patient tolerance | Accuracy reduced in hypothermic or underweight patients; limited to axillary temperature only |
| Du et al. | 2022 | China | Prospective Experimental Study | 64 diabetic nephropathy patients receiving home nursing intervention | PDCA (Plan-Do-Check-Action) home nursing strategy evaluated with fMRI under AI-based FCM | Improved clinical effectiveness; higher quality of life; increased patient satisfaction | N/A | N/A | PDCA nursing significantly improved curative effects, patient satisfaction, and quality of life; AI-based FCM | Small sample size; no consideration of long-term impacts on renal and glycemic indicators |

| | | | | | | | | | | |
|---|---|---|---|---|---|---|---|---|---|---|
| | | | | | clustering algorithm vs. routine home nursing | | | | improved fMRI analysis accuracy and efficiency | |
| Ping Yin, Hongli Wang | 2022 | China | Prospective Controlled Study | 60 postpartum women with pelvic organ prolapse (POP-Q grade I–II) | Pelvic floor rehabilitation training (PFMT, biofeedback, electrical stimulation) evaluated via AI-processed ultrasound imaging vs. standard Kegel exercises | Improved pelvic floor muscle strength; reduced prolapse severity; enhanced quality of life | N/A | N/A | Comprehensive pelvic floor training significantly improved anorectal functions and QoL; AI-enhanced ultrasound provided clearer imaging | Small sample size; short follow-up period; limited to mild and moderate POP cases |
| Seibert et al. | 2023 | Germany | Exploratory Sequential Mixed Methods Study | Stakeholders in AI and nursing care (21 workshop participants, 14 interviewees, 53 survey respondents, 80 datathon participants) | Exploration of needs, challenges, and opportunities for AI in nursing care | N/A | Optimized staff scheduling and workflows | Identified need for AI solutions in care assessment, planning, decision support | Identified needs for AI solutions in care assessment, planning, decision support, education, and resource management; barriers include data quality, regulatory issues, and ethical concerns | Limited to German context; short study duration; lack of data saturation due to exploratory nature |
| Racine et al.. | 2024 | Canada and UK | Multisite Qualitative Study | 20 health care professionals (HCPs) and 20 parents of preterm infants in NICUs at two tertiary hospitals | Use of AI for pain monitoring in neonatal intensive care units (NICU) | Enhanced awareness and standardization of pain assessment | N/A | N/A | AI as a supportive tool; ethical concerns; need for transparency and proper training | Limited to two NICUs in high-resource countries; perspectives were hypothetical due to lack of prior exposure to AI tools |
| Chen et al. | 2022 | China | Randomized Controlled Trial | 120 patients with CKD stages 3-5 using hospital-to-home (H2H) care | AI-based “Internet + Hospital-to-Home (H2H)” nutritional nursing model combined with AI-enhanced CT imaging vs. | Improved nutritional status; better biochemical markers; enhanced renal blood flow; higher patient satisfaction | Optimized monitoring and individualized care through AI-enhanced CT imaging | N/A | AI-enhanced H2H model significantly improved nutritional status, biochemical markers, renal blood flow, and patient | Single-center study; no separate analysis for CKD stages; results may not generalize beyond the study setting |

| | | | | | | | | | | |
|---|---|---|---|---|---|---|---|---|---|---|
| | | | | | conventional nutritional nursing model | | | | satisfaction compared to standard care | |
| Hassan et al. | 2024 | Egypt | Interpretive Phenomenological Analysis (IPA) | 10 critical care nurse leaders in ICUs across four hospitals | Exploration of AI integration in ICU care, including impact on practice, roles, trust, and ethical considerations | Enhanced clinical decision-making with AI support | Initial workload increased due to training and troubleshooting AI systems | Improved focus on patient-centered care by offloading routine tasks | AI enhances decision-making; concerns about overreliance and ethical biases; need for transparency and ongoing training | Small sample size; limited to leadership roles; bedside nurses' perspectives not captured |
| Sommer et al. | 2024 | Germany | Cross-Sectional Online Survey | 114 nurses from various care settings in Bavaria | Survey on nurses' perceptions, knowledge, and experiences of AI in nursing care | N/A | Potential for workflow optimization and administrative burden reduction | Positive attitudes towards AI as efficiency enhancer; concerns about job displacement | 25.2% self-identified as AI experts; 65.7% viewed AI positively for reducing workload; concerns include job loss and implementation costs | Small sample size; limited to Bavaria; potential sampling bias; lack of longitudinal data |
| Rosa et al. | 2024 | Brazil | Retrospective Observational Study | 43,871 patient assessments using Perroca's Patient Classification System (PCS) | Development of an AI-based predictive nursing workload classifier using machine learning (Random Forest algorithm) | N/A | Automated and optimized workload assessments; data-driven staff allocation | N/A | AI-based predictive model achieved 72% accuracy (AUC = 82%); key workload predictors identified include bed baths, fall risk, wound care protocols | Single-center study; lower performance for semi-intensive and intensive care classes; results may not generalize to other settings |
| Zhang et al. | 2022 | China | Randomized Controlled Study | 88 rectal cancer patients undergoing anus-preserving surgery | Comprehensive pelvic floor muscle rehabilitation exercises + AI-based MRI vs. standard Kegel exercises | Improved anorectal function; enhanced quality of life | N/A | N/A | Comprehensive pelvic floor training significantly improved anorectal functions and QoL; AI-enhanced MRI provided clearer imaging for diagnosis and assessment | Single-center study; small sample size; short intervention duration (3 months); no long-term follow-up |
