# Supplementary material for: Artificial intelligence in nursing: an integrative review of clinical and operational impacts
Source: Front Digit Health. 2025 Mar 7;7:1552372. doi: 10.3389/fdgth.2025.1552372 (PMC11926144; doi:10.3389/fdgth.2025.1552372)
Supplement: Supplementary file 2 [file Table2.pdf]

Supplementary Table 2 : Risk of Bias

Authors	Bias due to Confounding	Bias in Selection of Participants	Bias in Classification of Interventions	Bias due to Deviations from Intended Interventions	Bias due to Missing Data	Bias in Measurement of Outcomes	Bias in Selection of Reported Results	Overall Risk of Bias
Zhang et al. (2022)	Moderate	Low	Low	Low	Low	Low	Moderate	Moderate
Rosa et al. (2024)	Moderate	Low	Low	Low	Low	Moderate	Moderate	Moderate
Sommer et al. (2024)	Moderate	Moderate	Low	Low	Low	Moderate	Moderate	Moderate
Hassan et al. (2024)	Moderate	Moderate	Low	Low	Low	Moderate	Moderate	Moderate
Chen et al. (2022)	Moderate	Low	Low	Low	Low	Moderate	Moderate	Moderate
Racine et al. (2024)	Moderate	Moderate	Low	Not applicable	Low	Moderate	Moderate	Moderate
Seibert et al. (2023)	Moderate	Moderate	Not Applicable	Not Applicable	Low	Moderate	Moderate	Moderate
Yin et al. (2022)	Moderate	Low	Low	Low	Low	Moderate	Moderate	Moderate
Du et al. (2022)	Moderate	Low	Not Applicable	Not Applicable	Low	Moderate	Moderate	Moderate
Liu et al. (2020)	Moderate	Low	Low	Not Applicable	Low	Moderate	Moderate	Moderate
Marcuzzi et al. (2023)	Moderate	Low	Low	Low	Low	Moderate	Moderate	Moderate
Jiang et al. (2022)	Moderate	Low	Low	Not Applicable	Low	Moderate	Moderate	Moderate

Cho et al. (2024)	Moderate	Moderate	Not Applicable	Not Applicable	Low	Moderate	Moderate	Moderate
Xu et al. (2022)	Moderate	Low	Low	Low	Low	Moderate	Moderate	Moderate
Bian et al. (2020)	Moderate	Low	Low	Low	Low	Moderate	Moderate	Moderate
Rony et al. (2024)	Moderate	Moderate	Not Applicable	Not Applicable	Low	Moderate	Moderate	Moderate
Hong et al. (2021)	Moderate	Moderate	Low	Low	Low	Moderate	Moderate	Moderate
Alruwaili et al. (2024)	Moderate	Moderate	Not Applicable	Not Applicable	Low	Moderate	Moderate	Moderate
