# Supplementary material for: Artificial intelligence in nursing: an integrative review of clinical and operational impacts
Source: Front Digit Health. 2025 Mar 7;7:1552372. doi: 10.3389/fdgth.2025.1552372 (PMC11926144; doi:10.3389/fdgth.2025.1552372)
Supplement: Supplementary file 3 [file Table3.pdf]

Supplementary Table 3: MMAT Quality Assessment of Included Studies

No.	Authors et al. (Year)	Study Title	Study Type	Criterion 1: Clear & Relevant Questions	Criterion 2: Appropriate Sampling Strategy	Criterion 3: Representative Sample	Criterion 4: Appropriate Measurements	Criterion 5: Acceptable Response/Follow-Up Rate	Final MMAT Score
1	Alruwaili et al. (2024)	Assessment of Nurses' Awareness and Attitudes Toward AI in Clinical Practice	Cross-Sectional Survey	Yes	Yes	Partially	Yes	Yes	4/5
2	Hong et al. (2021)	Application of Artificial Intelligence in Emergency Nursing of Patients with Chronic Obstructive Pulmonary Disease	Randomized Controlled Trial (RCT)	Yes	Unclear	Partially	Yes	Partially	3/5
3	Rony et al. (2024)	Artificial Intelligence in Future Nursing Care: Exploring Perspectives of Nursing Professionals	Qualitative Study	Yes	Yes	Yes	Yes	Yes	5/5
4	Bian et al. (2020)	Artificial Intelligence–Assisted System in Postoperative Follow-up of Orthopedic Patients	Quantitative Descriptive Study	Yes	Yes	Yes	Yes	Yes	5/5
5	Xu et al. (2022)	Cerebral Angiography under AI Algorithm in	Randomized Controlled Trial (RCT)	Yes	Yes	Yes	Yes	Yes	5/5

		Nursing Cooperation Plan for Intracranial Aneurysm Patients in Craniotomy Clipping							
6	Cho et al. (2024)	Development of an AI-Based Tailored Mobile Intervention for Nurse Burnout	Quantitative Descriptive Study	Yes	Yes	Yes	Yes	Yes	5/5
7	Jiang et al. (2022)	Diagnosis and Nursing Intervention of Gynecological Ovarian Endometriosis with AI-Based MRI Algorithm	Randomized Controlled Trial (RCT)	Yes	Yes	Yes	Yes	Yes	5/5
8	Marcuzzi et al. (2023)	Effect of an AI-Based Self-Management App on Musculoskeletal Health in Patients with Neck and/or Low Back Pain Referred to Specialist Care	Randomized Controlled Trial (RCT)	Yes	Yes	Yes	Yes	Yes	5/5
9	Liu et al (2020)	Evaluation of a Wearable Wireless Device with AI, iThermonitor WT705, for Continuous Temperature Monitoring in Surgical Wards	Observational Study	Yes	Yes	Yes	Yes	Yes	5/5

10	Du et al. (2022)	Evaluation of Functional MRI under AI Algorithm on PDCA Home Nursing for Patients with Diabetic Nephropathy	Randomized Controlled Trial (RCT)	Yes	Yes	Yes	Yes	Yes	5/5
11	Yin et al. (2022)	Evaluation of Nursing Effect of Pelvic Floor Rehabilitation Training on Pelvic Organ Prolapse in Postpartum Pregnant Women under AI-Based Ultrasound Imaging	Randomized Controlled Trial (RCT)	Yes	Yes	Yes	Yes	Yes	5/5
12	Seibert et al. (2023)	Exploring Needs and Challenges for AI in Nursing Care – Results of an Explorative Sequential Mixed Methods Study	Mixed Methods Study	Yes	Yes	Yes	Yes	Yes	5/5
13	Racine et al. (2024)	Health Care Professionals' and Parents' Perspectives on the Use of AI for Pain Monitoring in the Neonatal Intensive Care Unit	Qualitative Study	Yes	Yes	Yes	Yes	Yes	5/5
14	Chen et al. (2022)	Implementation of Hospital-to-Home Model for	Randomized Controlled Trial (RCT)	Yes	Yes	Yes	Yes	Yes	5/5

		Nutritional Nursing Management of Patients with Chronic Kidney Disease Using AI Algorithm Combined with CT Internet							
15	Hassan et al. (2024)	Leading with AI in Critical Care Nursing: Challenges, Opportunities, and the Human Factor	Qualitative Study	Yes	Yes	Yes	Yes	Yes	5/5
16	Sommer et al. (2024)	Nurses' Perceptions, Experience, and Knowledge Regarding AI: Results from a Cross-Sectional Online Survey in Germany	Cross-Sectional Survey	Yes	Yes	Partially	Yes	Yes	4/5
17	Rosa et al. (2024)	Nursing Workload: Use of AI to Develop a Classifier Model	Quantitative Descriptive Study	Yes	Yes	Yes	Yes	Yes	5/5
18	Zhang et al. (2022)	Evaluation of Nursing Effects of Pelvic Floor Muscle Rehabilitation Exercise on Rectal Cancer Patients Receiving Anus-Preserving	Randomized Controlled Trial (RCT)	Yes	Yes	Yes	Yes	Yes	5/5

		Operation by AI-Based MRI							
